# Supplementary material for: A consideration of publication-derived immune-related associations in Coronavirus and related lung damaging diseases
Source: J Transl Med. 2020 Aug 3;18:297. doi: 10.1186/s12967-020-02472-z (PMC7397454; doi:10.1186/s12967-020-02472-z)
Supplement: Supplementary file 1 — Additional file 1: a) Occurrences of cytokine MeSh terms in the four corpuses. b) Occurrences of cell-type MeSh terms in the four corpuses. c) Occurrences of disease MeSh terms in the four corpuses (only diseases with >1 occurrences are shown). [file 12967_2020_2472_MOESM1_ESM.docx]

Additional file 1

**a) Occurrences of cytokine MeSh terms in the four corpuses**

|  | SARS  (raw count) | Coronavirus (raw count) | H5N1  (raw count) | COVID-19 (raw count) | SARS (%) | Coronavirus (%) | H5N1 (%) | COVID-19 (%) |
| --- | --- | --- | --- | --- | --- | --- | --- | --- |
| INTERFERON-GAMMA | 34 | 174 | 62 | 4 | 0.757 | 1.774 | 1.038 | 0.066 |
| INTERFERON-BETA | 13 | 59 | 38 | 6 | 0.289 | 0.601 | 0.636 | 0.100 |
| TUMOR NECROSIS FACTOR-ALPHA | 18 | 61 | 36 | 6 | 0.401 | 0.622 | 0.603 | 0.100 |
| INTERLEUKIN-6 | 14 | 35 | 29 | 52 | 0.312 | 0.357 | 0.485 | 0.862 |
| INTERFERON-ALPHA | 28 | 67 | 22 | 11 | 0.623 | 0.683 | 0.368 | 0.182 |
| INTERFERON TYPE I | 24 | 78 | 20 | 7 | 0.534 | 0.795 | 0.335 | 0.116 |
| INTERLEUKIN-4 | 9 | 21 | 14 | 1 | 0.200 | 0.214 | 0.234 | 0.017 |
| CHEMOKINE CXCL10 | 12 | 19 | 11 | 0 | 0.267 | 0.194 | 0.184 | 0.000 |
| INTERLEUKIN-2 | 6 | 22 | 6 | 2 | 0.134 | 0.224 | 0.100 | 0.033 |
| INTERLEUKIN-8 | 10 | 15 | 6 | 1 | 0.223 | 0.153 | 0.100 | 0.017 |
| INTERLEUKIN-10 | 6 | 14 | 5 | 5 | 0.134 | 0.143 | 0.084 | 0.083 |
| CHEMOKINE CCL2 | 7 | 10 | 4 | 1 | 0.156 | 0.102 | 0.067 | 0.017 |
| GRANULOCYTE-MACROPHAGE COLONY-STIMULATING FACTOR | 0 | 5 | 4 | 3 | 0.000 | 0.051 | 0.067 | 0.050 |
| INTERLEUKIN-12 | 2 | 16 | 4 | 0 | 0.045 | 0.163 | 0.067 | 0.000 |
| INTERLEUKIN-1BETA | 1 | 12 | 4 | 1 | 0.022 | 0.122 | 0.067 | 0.017 |
| TRANSFORMING GROWTH FACTOR BETA | 6 | 4 | 4 | 1 | 0.134 | 0.041 | 0.067 | 0.017 |
| INTERLEUKIN-12 SUBUNIT P40 | 0 | 2 | 3 | 0 | 0.000 | 0.020 | 0.050 | 0.000 |
| INTERLEUKIN-18 | 0 | 7 | 3 | 0 | 0.000 | 0.071 | 0.050 | 0.000 |
| CHEMOKINE CCL3 | 0 | 7 | 2 | 0 | 0.000 | 0.071 | 0.033 | 0.000 |
| CHEMOKINE CCL5 | 4 | 10 | 2 | 1 | 0.089 | 0.102 | 0.033 | 0.017 |
| INTERLEUKIN-1 | 4 | 13 | 2 | 4 | 0.089 | 0.133 | 0.033 | 0.066 |
| INTERLEUKIN-17 | 0 | 8 | 2 | 3 | 0.000 | 0.082 | 0.033 | 0.050 |
| TRANSFORMING GROWTH FACTOR BETA1 | 1 | 2 | 2 | 0 | 0.022 | 0.020 | 0.033 | 0.000 |
| CHEMOKINE CCL19 | 0 | 1 | 1 | 0 | 0.000 | 0.010 | 0.017 | 0.000 |
| CHEMOKINE CCL21 | 0 | 1 | 1 | 0 | 0.000 | 0.010 | 0.017 | 0.000 |
| CHEMOKINE CCL4 | 0 | 8 | 1 | 0 | 0.000 | 0.082 | 0.017 | 0.000 |
| CHEMOKINE CCL8 | 0 | 0 | 1 | 0 | 0.000 | 0.000 | 0.017 | 0.000 |
| CHEMOKINE CXCL13 | 0 | 0 | 1 | 0 | 0.000 | 0.000 | 0.017 | 0.000 |
| CHEMOKINE CXCL6 | 0 | 0 | 1 | 0 | 0.000 | 0.000 | 0.017 | 0.000 |
| CHEMOKINES, CXC | 8 | 15 | 1 | 0 | 0.178 | 0.153 | 0.017 | 0.000 |
| ERYTHROPOIETIN | 0 | 0 | 1 | 2 | 0.000 | 0.000 | 0.017 | 0.033 |
| GRANULOCYTE COLONY-STIMULATING FACTOR | 0 | 2 | 1 | 1 | 0.000 | 0.020 | 0.017 | 0.017 |
| HEPATOCYTE GROWTH FACTOR | 0 | 0 | 1 | 0 | 0.000 | 0.000 | 0.017 | 0.000 |
| INTERLEUKIN-15 | 0 | 6 | 1 | 0 | 0.000 | 0.061 | 0.017 | 0.000 |
| INTERLEUKIN-16 | 0 | 0 | 1 | 0 | 0.000 | 0.000 | 0.017 | 0.000 |
| MACROPHAGE INFLAMMATORY PROTEINS | 0 | 5 | 1 | 0 | 0.000 | 0.051 | 0.017 | 0.000 |
| MACROPHAGE MIGRATION-INHIBITORY FACTORS | 0 | 0 | 1 | 0 | 0.000 | 0.000 | 0.017 | 0.000 |
| TUMOR NECROSIS FACTORS | 1 | 0 | 1 | 0 | 0.022 | 0.000 | 0.017 | 0.000 |
| B-CELL ACTIVATING FACTOR | 0 | 1 | 0 | 0 | 0.000 | 0.010 | 0.000 | 0.000 |
| BETA-THROMBOGLOBULIN | 1 | 0 | 0 | 0 | 0.022 | 0.000 | 0.000 | 0.000 |
| CHEMOKINE CCL1 | 0 | 0 | 0 | 0 | 0.000 | 0.000 | 0.000 | 0.000 |
| CHEMOKINE CCL11 | 0 | 0 | 0 | 0 | 0.000 | 0.000 | 0.000 | 0.000 |
| CHEMOKINE CCL17 | 0 | 0 | 0 | 0 | 0.000 | 0.000 | 0.000 | 0.000 |
| CHEMOKINE CCL20 | 0 | 0 | 0 | 0 | 0.000 | 0.000 | 0.000 | 0.000 |
| CHEMOKINE CCL22 | 0 | 0 | 0 | 0 | 0.000 | 0.000 | 0.000 | 0.000 |
| CHEMOKINE CCL24 | 0 | 0 | 0 | 0 | 0.000 | 0.000 | 0.000 | 0.000 |
| CHEMOKINE CCL27 | 0 | 0 | 0 | 0 | 0.000 | 0.000 | 0.000 | 0.000 |
| CHEMOKINE CCL7 | 0 | 0 | 0 | 0 | 0.000 | 0.000 | 0.000 | 0.000 |
| CHEMOKINE CX3CL1 | 0 | 0 | 0 | 0 | 0.000 | 0.000 | 0.000 | 0.000 |
| CHEMOKINE CXCL1 | 0 | 3 | 0 | 0 | 0.000 | 0.031 | 0.000 | 0.000 |
| CHEMOKINE CXCL11 | 1 | 1 | 0 | 0 | 0.022 | 0.010 | 0.000 | 0.000 |
| CHEMOKINE CXCL12 | 0 | 1 | 0 | 0 | 0.000 | 0.010 | 0.000 | 0.000 |
| CHEMOKINE CXCL2 | 0 | 3 | 0 | 0 | 0.000 | 0.031 | 0.000 | 0.000 |
| CHEMOKINE CXCL5 | 0 | 1 | 0 | 0 | 0.000 | 0.010 | 0.000 | 0.000 |
| CHEMOKINE CXCL9 | 4 | 5 | 0 | 0 | 0.089 | 0.051 | 0.000 | 0.000 |
| CHEMOKINES, C | 0 | 0 | 0 | 0 | 0.000 | 0.000 | 0.000 | 0.000 |
| CHEMOKINES, CC | 1 | 6 | 0 | 0 | 0.022 | 0.061 | 0.000 | 0.000 |
| CHEMOKINES, CX3C | 0 | 0 | 0 | 0 | 0.000 | 0.000 | 0.000 | 0.000 |
| GROWTH DIFFERENTIATION FACTOR 15 | 0 | 0 | 0 | 0 | 0.000 | 0.000 | 0.000 | 0.000 |
| INTERLEUKIN 1 RECEPTOR ANTAGONIST PROTEIN | 0 | 1 | 0 | 1 | 0.000 | 0.010 | 0.000 | 0.017 |
| INTERLEUKIN-11 | 0 | 3 | 0 | 0 | 0.000 | 0.031 | 0.000 | 0.000 |
| INTERLEUKIN-12 SUBUNIT P35 | 0 | 0 | 0 | 0 | 0.000 | 0.000 | 0.000 | 0.000 |
| INTERLEUKIN-13 | 1 | 0 | 0 | 0 | 0.022 | 0.000 | 0.000 | 0.000 |
| INTERLEUKIN-1ALPHA | 0 | 1 | 0 | 0 | 0.000 | 0.010 | 0.000 | 0.000 |
| INTERLEUKIN-23 | 0 | 2 | 0 | 2 | 0.000 | 0.020 | 0.000 | 0.033 |
| INTERLEUKIN-23 SUBUNIT P19 | 0 | 0 | 0 | 0 | 0.000 | 0.000 | 0.000 | 0.000 |
| INTERLEUKIN-3 | 0 | 1 | 0 | 0 | 0.000 | 0.010 | 0.000 | 0.000 |
| INTERLEUKIN-5 | 0 | 1 | 0 | 0 | 0.000 | 0.010 | 0.000 | 0.000 |
| INTERLEUKIN-7 | 0 | 1 | 0 | 0 | 0.000 | 0.010 | 0.000 | 0.000 |
| INTERLEUKIN-9 | 0 | 0 | 0 | 0 | 0.000 | 0.000 | 0.000 | 0.000 |
| LEUKEMIA INHIBITORY FACTOR | 0 | 0 | 0 | 0 | 0.000 | 0.000 | 0.000 | 0.000 |
| LEUKOCYTE MIGRATION-INHIBITORY FACTORS | 0 | 0 | 0 | 0 | 0.000 | 0.000 | 0.000 | 0.000 |
| LYMPHOTOXIN-ALPHA | 0 | 0 | 0 | 0 | 0.000 | 0.000 | 0.000 | 0.000 |
| MACROPHAGE COLONY-STIMULATING FACTOR | 0 | 0 | 0 | 0 | 0.000 | 0.000 | 0.000 | 0.000 |
| MACROPHAGE-ACTIVATING FACTORS | 0 | 0 | 0 | 0 | 0.000 | 0.000 | 0.000 | 0.000 |
| MONOCYTE CHEMOATTRACTANT PROTEINS | 0 | 0 | 0 | 0 | 0.000 | 0.000 | 0.000 | 0.000 |
| ONCOSTATIN M | 0 | 0 | 0 | 0 | 0.000 | 0.000 | 0.000 | 0.000 |
| OSTEOPONTIN | 0 | 0 | 0 | 0 | 0.000 | 0.000 | 0.000 | 0.000 |
| PLATELET FACTOR 4 | 1 | 0 | 0 | 0 | 0.022 | 0.000 | 0.000 | 0.000 |
| STEM CELL FACTOR | 0 | 0 | 0 | 0 | 0.000 | 0.000 | 0.000 | 0.000 |
| SUPPRESSOR FACTORS, IMMUNOLOGIC | 0 | 0 | 0 | 0 | 0.000 | 0.000 | 0.000 | 0.000 |
| THROMBOPOIETIN | 1 | 0 | 0 | 0 | 0.022 | 0.000 | 0.000 | 0.000 |
| TRANSFER FACTOR | 0 | 0 | 0 | 0 | 0.000 | 0.000 | 0.000 | 0.000 |
| TRANSFORMING GROWTH FACTOR BETA2 | 0 | 0 | 0 | 0 | 0.000 | 0.000 | 0.000 | 0.000 |
| TRANSFORMING GROWTH FACTOR BETA3 | 0 | 0 | 0 | 0 | 0.000 | 0.000 | 0.000 | 0.000 |

**b) Occurrences of cell-type MeSh terms in the four corpuses**

|  | SARS  (raw count) | Coronavirus (raw count) | H5N1  (raw count) | COVID-19 (raw count) | SARS (%) | Coronavirus (%) | H5N1 (%) | COVID-19 (%) |
| --- | --- | --- | --- | --- | --- | --- | --- | --- |
| MACROPHAGES | 20 | 205 | 66 | 14 | 0.445 | 2.090 | 1.105 | 0.232 |
| T-LYMPHOCYTES | 31 | 159 | 77 | 27 | 0.690 | 1.621 | 1.289 | 0.448 |
| CD8-POSITIVE T-LYMPHOCYTES | 42 | 122 | 57 | 14 | 0.935 | 1.244 | 0.954 | 0.232 |
| CD4-POSITIVE T-LYMPHOCYTES | 31 | 91 | 39 | 7 | 0.690 | 0.928 | 0.653 | 0.116 |
| B-LYMPHOCYTES | 16 | 60 | 38 | 9 | 0.356 | 0.612 | 0.636 | 0.149 |
| DENDRITIC CELLS | 16 | 60 | 30 | 3 | 0.356 | 0.612 | 0.502 | 0.050 |
| T-LYMPHOCYTES, CYTOTOXIC | 21 | 59 | 20 | 1 | 0.467 | 0.601 | 0.335 | 0.017 |
| MONOCYTES | 12 | 48 | 15 | 6 | 0.267 | 0.489 | 0.251 | 0.100 |
| T-LYMPHOCYTE SUBSETS | 25 | 46 | 5 | 6 | 0.556 | 0.469 | 0.084 | 0.100 |
| LYMPHOCYTES | 8 | 44 | 11 | 16 | 0.178 | 0.449 | 0.184 | 0.265 |
| LEUKOCYTES, MONONUCLEAR | 10 | 42 | 25 | 2 | 0.223 | 0.428 | 0.418 | 0.033 |
| KILLER CELLS, NATURAL | 5 | 35 | 14 | 8 | 0.111 | 0.357 | 0.234 | 0.133 |
| TH1 CELLS | 6 | 27 | 15 | 1 | 0.134 | 0.275 | 0.251 | 0.017 |
| MACROPHAGES, PERITONEAL | 1 | 25 | 0 | 0 | 0.022 | 0.255 | 0.000 | 0.000 |
| ERYTHROCYTES | 3 | 25 | 18 | 0 | 0.067 | 0.255 | 0.301 | 0.000 |
| LEUKOCYTES | 7 | 22 | 4 | 3 | 0.156 | 0.224 | 0.067 | 0.050 |
| BONE MARROW | 4 | 18 | 5 | 1 | 0.089 | 0.183 | 0.084 | 0.017 |
| NEUTROPHILS | 8 | 17 | 18 | 26 | 0.178 | 0.173 | 0.301 | 0.431 |
| ANTIBODY-PRODUCING CELLS | 0 | 16 | 2 | 0 | 0.000 | 0.163 | 0.033 | 0.000 |
| T-LYMPHOCYTES, HELPER-INDUCER | 1 | 15 | 3 | 1 | 0.022 | 0.153 | 0.050 | 0.017 |
| MACROPHAGES, ALVEOLAR | 7 | 14 | 10 | 2 | 0.156 | 0.143 | 0.167 | 0.033 |
| T-LYMPHOCYTES, REGULATORY | 0 | 12 | 2 | 1 | 0.000 | 0.122 | 0.033 | 0.017 |
| ANTIGEN-PRESENTING CELLS | 1 | 11 | 3 | 0 | 0.022 | 0.112 | 0.050 | 0.000 |
| TH2 CELLS | 7 | 11 | 6 | 0 | 0.156 | 0.112 | 0.100 | 0.000 |
| RETICULOCYTES | 0 | 10 | 0 | 0 | 0.000 | 0.102 | 0.000 | 0.000 |
| HEMATOPOIETIC STEM CELLS | 2 | 6 | 2 | 2 | 0.045 | 0.061 | 0.033 | 0.033 |
| BASOPHILS | 0 | 4 | 1 | 0 | 0.000 | 0.041 | 0.017 | 0.000 |
| MONONUCLEAR PHAGOCYTE SYSTEM | 0 | 4 | 0 | 0 | 0.000 | 0.041 | 0.000 | 0.000 |
| BLOOD PLATELETS | 0 | 3 | 2 | 4 | 0.000 | 0.031 | 0.033 | 0.066 |
| TH17 CELLS | 0 | 3 | 0 | 2 | 0.000 | 0.031 | 0.000 | 0.033 |
| B-LYMPHOCYTE SUBSETS | 2 | 3 | 0 | 0 | 0.045 | 0.031 | 0.000 | 0.000 |
| PHAGOCYTES | 1 | 2 | 0 | 0 | 0.022 | 0.020 | 0.000 | 0.000 |
| MAST CELLS | 0 | 1 | 2 | 0 | 0.000 | 0.010 | 0.033 | 0.000 |
| NATURAL KILLER T-CELLS | 0 | 1 | 1 | 0 | 0.000 | 0.010 | 0.017 | 0.000 |
| GRANULOCYTES | 1 | 1 | 1 | 1 | 0.022 | 0.010 | 0.017 | 0.017 |
| MEGAKARYOCYTES | 1 | 1 | 0 | 3 | 0.022 | 0.010 | 0.000 | 0.050 |
| EOSINOPHILS | 3 | 1 | 1 | 1 | 0.067 | 0.010 | 0.017 | 0.017 |
| ACANTHOCYTES | 0 | 0 | 0 | 0 | 0.000 | 0.000 | 0.000 | 0.000 |
| B-LYMPHOCYTES, REGULATORY | 0 | 0 | 0 | 0 | 0.000 | 0.000 | 0.000 | 0.000 |
| CYTOKINE-INDUCED KILLER CELLS | 0 | 0 | 0 | 0 | 0.000 | 0.000 | 0.000 | 0.000 |
| DENDRITIC CELLS, FOLLICULAR | 0 | 0 | 0 | 0 | 0.000 | 0.000 | 0.000 | 0.000 |
| ERYTHROBLASTS | 0 | 0 | 0 | 0 | 0.000 | 0.000 | 0.000 | 0.000 |
| ERYTHROCYTES, ABNORMAL | 0 | 0 | 0 | 0 | 0.000 | 0.000 | 0.000 | 0.000 |
| ERYTHROID PRECURSOR CELLS | 0 | 0 | 0 | 0 | 0.000 | 0.000 | 0.000 | 0.000 |
| GIANT CELLS, FOREIGN-BODY | 0 | 0 | 0 | 0 | 0.000 | 0.000 | 0.000 | 0.000 |
| GRANULOCYTE PRECURSOR CELLS | 0 | 0 | 0 | 0 | 0.000 | 0.000 | 0.000 | 0.000 |
| GRANULOCYTE-MACROPHAGE PROGENITOR CELLS | 0 | 0 | 0 | 0 | 0.000 | 0.000 | 0.000 | 0.000 |
| HEMOCYTES | 0 | 0 | 0 | 1 | 0.000 | 0.000 | 0.000 | 0.017 |
| KILLER CELLS, LYMPHOKINE-ACTIVATED | 0 | 0 | 0 | 0 | 0.000 | 0.000 | 0.000 | 0.000 |
| LANGERHANS CELLS | 0 | 0 | 1 | 0 | 0.000 | 0.000 | 0.017 | 0.000 |
| LYMPHOCYTES, NULL | 0 | 0 | 0 | 0 | 0.000 | 0.000 | 0.000 | 0.000 |
| LYMPHOCYTES, TUMOR-INFILTRATING | 0 | 0 | 0 | 0 | 0.000 | 0.000 | 0.000 | 0.000 |
| LYMPHOID PROGENITOR CELLS | 0 | 0 | 0 | 0 | 0.000 | 0.000 | 0.000 | 0.000 |
| MEGAKARYOCYTE PROGENITOR CELLS | 0 | 0 | 0 | 0 | 0.000 | 0.000 | 0.000 | 0.000 |
| MEGAKARYOCYTE-ERYTHROID PROGENITOR CELLS | 0 | 0 | 0 | 0 | 0.000 | 0.000 | 0.000 | 0.000 |
| MEGALOBLASTS | 0 | 0 | 0 | 0 | 0.000 | 0.000 | 0.000 | 0.000 |
| MONOCYTE-MACROPHAGE PRECURSOR CELLS | 0 | 0 | 0 | 0 | 0.000 | 0.000 | 0.000 | 0.000 |
| MONOCYTES, ACTIVATED KILLER | 0 | 0 | 0 | 0 | 0.000 | 0.000 | 0.000 | 0.000 |
| MYELOID PROGENITOR CELLS | 0 | 0 | 0 | 0 | 0.000 | 0.000 | 0.000 | 0.000 |
| PERIPHERAL BLOOD MONONUCLEAR CELL | 0 | 0 | 0 | 0 | 0.000 | 0.000 | 0.000 | 0.000 |
| PERIPHERAL BLOOD MONONUCLEAR CELL | 0 | 0 | 0 | 0 | 0.000 | 0.000 | 0.000 | 0.000 |
| PRECURSOR CELLS, B-LYMPHOID | 0 | 0 | 0 | 0 | 0.000 | 0.000 | 0.000 | 0.000 |
| PRECURSOR CELLS, T-LYMPHOID | 0 | 0 | 0 | 0 | 0.000 | 0.000 | 0.000 | 0.000 |
| SPHEROCYTES | 0 | 0 | 0 | 0 | 0.000 | 0.000 | 0.000 | 0.000 |
| THYMOCYTES | 0 | 0 | 0 | 0 | 0.000 | 0.000 | 0.000 | 0.000 |

**c) Occurrences of disease MeSh terms in the four corpuses***

|  | SARS  (raw count) | Coronavirus (raw count) | H5N1  (raw count) | COVID-19 (raw count) | SARS (%) | Coronavirus (%) | H5N1 (%) | COVID-19 (%) |
| --- | --- | --- | --- | --- | --- | --- | --- | --- |
| SEVERE ACUTE RESPIRATORY SYNDROME | 4221 | 0 | 53 | 184 | 93.946 | 0.000 | 0.887 | 3.052 |
| PNEUMONIA | 58 | 27 | 18 | 24 | 1.291 | 0.275 | 0.301 | 0.398 |
| WEST NILE FEVER | 34 | 0 | 4 | 0 | 0.757 | 0.000 | 0.067 | 0.000 |
| ACQUIRED IMMUNODEFICIENCY SYNDROME | 31 | 3 | 1 | 2 | 0.690 | 0.031 | 0.017 | 0.033 |
| TUBERCULOSIS | 19 | 1 | 4 | 6 | 0.423 | 0.010 | 0.067 | 0.100 |
| LYMPHOPENIA | 18 | 9 | 5 | 29 | 0.401 | 0.092 | 0.084 | 0.481 |
| SMALLPOX | 18 | 1 | 4 | 0 | 0.401 | 0.010 | 0.067 | 0.000 |
| OSTEONECROSIS | 15 | 0 | 0 | 0 | 0.334 | 0.000 | 0.000 | 0.000 |
| PULMONARY FIBROSIS | 14 | 0 | 5 | 6 | 0.312 | 0.000 | 0.084 | 0.100 |
| DIARRHEA | 12 | 287 | 4 | 34 | 0.267 | 2.926 | 0.067 | 0.564 |
| PLAGUE | 12 | 1 | 2 | 4 | 0.267 | 0.010 | 0.033 | 0.066 |
| COMMON COLD | 11 | 25 | 2 | 1 | 0.245 | 0.255 | 0.033 | 0.017 |
| THROMBOCYTOPENIA | 11 | 4 | 1 | 9 | 0.245 | 0.041 | 0.017 | 0.149 |
| HEPATITIS C | 11 | 2 | 1 | 1 | 0.245 | 0.020 | 0.017 | 0.017 |
| LABORATORY INFECTIOUS DISEASE | 11 | 0 | 4 | 1 | 0.245 | 0.000 | 0.067 | 0.017 |
| POLIOMYELITIS | 9 | 2 | 3 | 1 | 0.200 | 0.020 | 0.050 | 0.017 |
| MALARIA | 9 | 0 | 3 | 3 | 0.200 | 0.000 | 0.050 | 0.050 |
| PNEUMOTHORAX | 9 | 0 | 0 | 6 | 0.200 | 0.000 | 0.000 | 0.100 |
| HEPATITIS B | 8 | 6 | 1 | 2 | 0.178 | 0.061 | 0.017 | 0.033 |
| SYNDROME | 7 | 29 | 0 | 0 | 0.156 | 0.296 | 0.000 | 0.000 |
| MONKEYPOX | 7 | 1 | 1 | 0 | 0.156 | 0.010 | 0.017 | 0.000 |
| CHOLERA | 7 | 0 | 2 | 3 | 0.156 | 0.000 | 0.033 | 0.050 |
| MEASLES | 6 | 4 | 6 | 2 | 0.134 | 0.041 | 0.100 | 0.033 |
| DIABETES MELLITUS | 6 | 2 | 0 | 44 | 0.134 | 0.020 | 0.000 | 0.730 |
| ASPERGILLOSIS | 6 | 0 | 0 | 0 | 0.134 | 0.000 | 0.000 | 0.000 |
| LEGIONNAIRES DISEASE | 6 | 0 | 0 | 2 | 0.134 | 0.000 | 0.000 | 0.033 |
| ASTHMA | 4 | 10 | 2 | 7 | 0.089 | 0.102 | 0.033 | 0.116 |
| HYPERTENSION | 4 | 4 | 0 | 63 | 0.089 | 0.041 | 0.000 | 1.045 |
| PULMONARY EDEMA | 4 | 1 | 2 | 2 | 0.089 | 0.010 | 0.033 | 0.033 |
| DISSEMINATED INTRAVASCULAR COAGULATION | 4 | 0 | 1 | 12 | 0.089 | 0.000 | 0.017 | 0.199 |
| MYOCARDIAL INFARCTION | 4 | 0 | 1 | 9 | 0.089 | 0.000 | 0.017 | 0.149 |
| SYSTEMIC INFLAMMATORY RESPONSE SYNDROME | 4 | 0 | 0 | 4 | 0.089 | 0.000 | 0.000 | 0.066 |
| GASTROENTERITIS | 3 | 33 | 1 | 2 | 0.067 | 0.336 | 0.017 | 0.033 |
| ANEMIA | 3 | 3 | 3 | 2 | 0.067 | 0.031 | 0.050 | 0.033 |
| YELLOW FEVER | 3 | 2 | 0 | 0 | 0.067 | 0.020 | 0.000 | 0.000 |
| CHICKENPOX | 3 | 1 | 0 | 0 | 0.067 | 0.010 | 0.000 | 0.000 |
| HYPERGLYCEMIA | 3 | 1 | 2 | 10 | 0.067 | 0.010 | 0.033 | 0.166 |
| MALNUTRITION | 3 | 1 | 1 | 5 | 0.067 | 0.010 | 0.017 | 0.083 |
| OBESITY | 3 | 1 | 0 | 34 | 0.067 | 0.010 | 0.000 | 0.564 |
| RIFT VALLEY FEVER | 3 | 1 | 0 | 0 | 0.067 | 0.010 | 0.000 | 0.000 |
| FIBROMYALGIA | 3 | 0 | 0 | 0 | 0.067 | 0.000 | 0.000 | 0.000 |
| HANTAVIRUS PULMONARY SYNDROME | 3 | 0 | 0 | 0 | 0.067 | 0.000 | 0.000 | 0.000 |
| LASSA FEVER | 3 | 0 | 0 | 1 | 0.067 | 0.000 | 0.000 | 0.017 |
| LEGIONELLOSIS | 3 | 0 | 0 | 0 | 0.067 | 0.000 | 0.000 | 0.000 |
| LYME DISEASE | 3 | 0 | 0 | 0 | 0.067 | 0.000 | 0.000 | 0.000 |
| MARBURG HEMORRHAGIC FEVER | 3 | 0 | 1 | 0 | 0.067 | 0.000 | 0.017 | 0.000 |
| HEPATITIS A | 2 | 34 | 0 | 0 | 0.045 | 0.347 | 0.000 | 0.000 |
| KAWASAKI DISEASE | 2 | 15 | 0 | 4 | 0.045 | 0.153 | 0.000 | 0.066 |
| HEPATITIS | 2 | 6 | 1 |  | 0.045 | 0.061 | 0.017 | 0.000 |
| LEUKOPENIA | 2 | 3 | 1 | 6 | 0.045 | 0.031 | 0.017 | 0.100 |
| RABIES | 2 | 2 | 3 | 0 | 0.045 | 0.020 | 0.050 | 0.000 |
| CEREBROVASCULAR DISEASE | 2 | 1 | 0 | 37 | 0.045 | 0.010 | 0.000 | 0.614 |
| CHIKUNGUNYA | 2 | 1 | 1 | 0 | 0.045 | 0.010 | 0.017 | 0.000 |
| CREUTZFELDT-JAKOB SYNDROME | 2 | 1 | 3 | 0 | 0.045 | 0.010 | 0.050 | 0.000 |
| LYMPHOMA | 2 | 1 | 0 | 3 | 0.045 | 0.010 | 0.000 | 0.050 |
| PANCREATITIS | 2 | 1 | 0 | 1 | 0.045 | 0.010 | 0.000 | 0.017 |
| PERTUSSIS | 2 | 1 | 1 | 0 | 0.045 | 0.010 | 0.017 | 0.000 |
| BRONCHOPNEUMONIA | 2 | 0 | 1 | 0 | 0.045 | 0.000 | 0.017 | 0.000 |
| CORONARY ARTERY DISEASE | 2 | 0 | 0 | 3 | 0.045 | 0.000 | 0.000 | 0.050 |
| PULMONARY EMBOLISM | 2 | 0 | 0 | 25 | 0.045 | 0.000 | 0.000 | 0.415 |
| Q FEVER | 2 | 0 | 0 | 0 | 0.045 | 0.000 | 0.000 | 0.000 |
| SCRUB TYPHUS | 2 | 0 | 0 | 0 | 0.045 | 0.000 | 0.000 | 0.000 |
| FELINE INFECTIOUS PERITONITIS | 1 | 243 | 0 | 0 | 0.022 | 2.477 | 0.000 | 0.000 |
| MULTIPLE SCLEROSIS | 1 | 48 | 0 | 9 | 0.022 | 0.489 | 0.000 | 0.149 |
| BRONCHITIS | 1 | 44 | 2 | 0 | 0.022 | 0.449 | 0.033 | 0.000 |
| ENCEPHALITIS | 1 | 37 | 4 | 3 | 0.022 | 0.377 | 0.067 | 0.050 |
| BRONCHIOLITIS | 1 | 10 | 0 | 0 | 0.022 | 0.102 | 0.000 | 0.000 |
| PERITONITIS | 1 | 8 | 0 | 0 | 0.022 | 0.082 | 0.000 | 0.000 |
| HERPES SIMPLEX | 1 | 5 | 2 | 0 | 0.022 | 0.051 | 0.033 | 0.000 |
| PHARYNGITIS | 1 | 5 | 0 | 11 | 0.022 | 0.051 | 0.000 | 0.182 |
| MYOCARDITIS | 1 | 4 | 3 | 12 | 0.022 | 0.041 | 0.050 | 0.199 |
| NEUTROPENIA | 1 | 3 | 0 | 1 | 0.022 | 0.031 | 0.000 | 0.017 |
| RETINAL DEGENERATION | 1 | 3 | 0 | 0 | 0.022 | 0.031 | 0.000 | 0.000 |
| ORCHITIS | 1 | 2 | 0 | 2 | 0.022 | 0.020 | 0.000 | 0.033 |
| AFRICAN SWINE FEVER | 1 | 1 | 1 | 0 | 0.022 | 0.010 | 0.017 | 0.000 |
| BIPOLAR DISORDER | 1 | 1 | 0 | 2 | 0.022 | 0.010 | 0.000 | 0.033 |
| BRUCELLOSIS | 1 | 1 | 1 | 0 | 0.022 | 0.010 | 0.017 | 0.000 |
| FUNGAL INFECTIOUS DISEASE | 1 | 1 | 1 | 2 | 0.022 | 0.010 | 0.017 | 0.033 |
| GASTROESOPHAGEAL REFLUX DISEASE | 1 | 1 | 0 | 0 | 0.022 | 0.010 | 0.000 | 0.000 |
| HYPERSENSITIVITY REACTION TYPE I DISEASE | 1 | 1 | 0 | 0 | 0.022 | 0.010 | 0.000 | 0.000 |
| RUBELLA | 1 | 1 | 0 | 2 | 0.022 | 0.010 | 0.000 | 0.033 |
| SCRAPIE | 1 | 1 | 0 | 0 | 0.022 | 0.010 | 0.000 | 0.000 |
| BILIRUBIN METABOLIC DISORDER | 1 | 0 | 0 | 0 | 0.022 | 0.000 | 0.000 | 0.000 |
| BOTULISM | 1 | 0 | 0 | 0 | 0.022 | 0.000 | 0.000 | 0.000 |
| CARCINOMA | 1 | 0 | 0 | 2 | 0.022 | 0.000 | 0.000 | 0.033 |
| CARDIOMYOPATHY | 1 | 0 | 0 | 7 | 0.022 | 0.000 | 0.000 | 0.116 |
| CEREBRAL INFARCTION | 1 | 0 | 0 | 2 | 0.022 | 0.000 | 0.000 | 0.033 |
| CYSTITIS | 1 | 0 | 0 | 0 | 0.022 | 0.000 | 0.000 | 0.000 |
| DECUBITUS ULCER | 1 | 0 | 0 | 2 | 0.022 | 0.000 | 0.000 | 0.033 |
| DISEASE | 1 | 0 | 0 | 0 | 0.022 | 0.000 | 0.000 | 0.000 |
| EPILEPSY SYNDROME | 1 | 0 | 0 | 4 | 0.022 | 0.000 | 0.000 | 0.066 |
| ERYTHEMA INFECTIOSUM | 1 | 0 | 0 | 0 | 0.022 | 0.000 | 0.000 | 0.000 |
| FACTOR VIII DEFICIENCY | 1 | 0 | 0 | 0 | 0.022 | 0.000 | 0.000 | 0.000 |
| GLANDERS | 1 | 0 | 0 | 0 | 0.022 | 0.000 | 0.000 | 0.000 |
| GLAUCOMA | 1 | 0 | 0 | 2 | 0.022 | 0.000 | 0.000 | 0.033 |
| GLUCOSE INTOLERANCE | 1 | 0 | 0 | 0 | 0.022 | 0.000 | 0.000 | 0.000 |
| GONORRHEA | 1 | 0 | 0 | 0 | 0.022 | 0.000 | 0.000 | 0.000 |
| HEMANGIOMA | 1 | 0 | 0 | 1 | 0.022 | 0.000 | 0.000 | 0.017 |
| HEMORRHAGIC FEVER WITH RENAL SYNDROME | 1 | 0 | 0 | 0 | 0.022 | 0.000 | 0.000 | 0.000 |
| HODGKINS LYMPHOMA | 1 | 0 | 0 | 2 | 0.022 | 0.000 | 0.000 | 0.033 |
| HYPOKALEMIA | 1 | 0 | 0 | 2 | 0.022 | 0.000 | 0.000 | 0.033 |
| INTESTINAL PERFORATION | 1 | 0 | 0 | 0 | 0.022 | 0.000 | 0.000 | 0.000 |
| KURU ENCEPHALOPATHY | 1 | 0 | 0 | 0 | 0.022 | 0.000 | 0.000 | 0.000 |
| LUNG DISEASE | 1 | 0 | 0 | 0 | 0.022 | 0.000 | 0.000 | 0.000 |
| MAGNESIUM DEFICIENCY | 1 | 0 | 0 | 0 | 0.022 | 0.000 | 0.000 | 0.000 |
| MELIOIDOSIS | 1 | 0 | 0 | 0 | 0.022 | 0.000 | 0.000 | 0.000 |
| MOTION SICKNESS | 1 | 0 | 0 | 0 | 0.022 | 0.000 | 0.000 | 0.000 |
| MYOSITIS | 1 | 0 | 2 | 2 | 0.022 | 0.000 | 0.033 | 0.033 |
| NASOPHARYNX CARCINOMA | 1 | 0 | 0 | 0 | 0.022 | 0.000 | 0.000 | 0.000 |
| OSTEOPOROSIS | 1 | 0 | 1 | 5 | 0.022 | 0.000 | 0.017 | 0.083 |
| PULMONARY EMPHYSEMA | 1 | 0 | 0 | 0 | 0.022 | 0.000 | 0.000 | 0.000 |
| PULMONARY EOSINOPHILIA | 1 | 0 | 0 | 0 | 0.022 | 0.000 | 0.000 | 0.000 |
| RELAPSING FEVER | 1 | 0 | 0 | 0 | 0.022 | 0.000 | 0.000 | 0.000 |
| ROCKY MOUNTAIN SPOTTED FEVER | 1 | 0 | 0 | 0 | 0.022 | 0.000 | 0.000 | 0.000 |
| SALMONELLA GASTROENTERITIS | 1 | 0 | 0 | 0 | 0.022 | 0.000 | 0.000 | 0.000 |
| STATUS EPILEPTICUS | 1 | 0 | 0 | 1 | 0.022 | 0.000 | 0.000 | 0.017 |
| SYPHILIS | 1 | 0 | 0 | 0 | 0.022 | 0.000 | 0.000 | 0.000 |
| TETANUS | 1 | 0 | 0 | 0 | 0.022 | 0.000 | 0.000 | 0.000 |
| THROMBOPHILIA | 1 | 0 | 0 | 22 | 0.022 | 0.000 | 0.000 | 0.365 |
| TOXOPLASMOSIS | 1 | 0 | 0 | 0 | 0.022 | 0.000 | 0.000 | 0.000 |
| TULAREMIA | 1 | 0 | 0 | 0 | 0.022 | 0.000 | 0.000 | 0.000 |
| TYPHOID FEVER | 1 | 0 | 1 | 2 | 0.022 | 0.000 | 0.017 | 0.033 |
| VERTEBROBASILAR INSUFFICIENCY | 1 | 0 | 0 | 0 | 0.022 | 0.000 | 0.000 | 0.000 |
| WATERHOUSE-FRIDERICHSEN SYNDROME | 1 | 0 | 0 | 0 | 0.022 | 0.000 | 0.000 | 0.000 |
| ENCEPHALOMYELITIS | 0 | 100 | 1 | 0 | 0.000 | 1.019 | 0.017 | 0.000 |
| NEWCASTLE DISEASE | 0 | 69 | 24 | 0 | 0.000 | 0.703 | 0.402 | 0.000 |
| ASTROCYTOMA | 0 | 28 | 0 | 0 | 0.000 | 0.285 | 0.000 | 0.000 |
| DYSENTERY | 0 | 26 | 0 | 0 | 0.000 | 0.265 | 0.000 | 0.000 |
| NEPHRITIS | 0 | 22 | 0 | 0 | 0.000 | 0.224 | 0.000 | 0.000 |
| PORCINE REPRODUCTIVE AND RESPIRATORY SYNDROME | 0 | 16 | 0 | 0 | 0.000 | 0.163 | 0.000 | 0.000 |
| CRYPTOSPORIDIOSIS | 0 | 12 | 1 | 0 | 0.000 | 0.122 | 0.017 | 0.000 |
| FELINE ACQUIRED IMMUNODEFICIENCY SYNDROME | 0 | 9 | 0 | 0 | 0.000 | 0.092 | 0.000 | 0.000 |
| BOVINE VIRUS DIARRHEA-MUCOSAL DISEASE | 0 | 7 | 0 | 0 | 0.000 | 0.071 | 0.000 | 0.000 |
| PSEUDORABIES | 0 | 7 | 0 | 0 | 0.000 | 0.071 | 0.000 | 0.000 |
| NEUROBLASTOMA | 0 | 6 | 0 | 0 | 0.000 | 0.061 | 0.000 | 0.000 |
| BOVINE RESPIRATORY DISEASE COMPLEX | 0 | 5 | 0 | 0 | 0.000 | 0.051 | 0.000 | 0.000 |
| CROUP | 0 | 5 | 0 | 0 | 0.000 | 0.051 | 0.000 | 0.000 |
| FELINE PANLEUKOPENIA | 0 | 5 | 0 | 0 | 0.000 | 0.051 | 0.000 | 0.000 |
| LYMPHOCYTIC CHORIOMENINGITIS | 0 | 5 | 0 | 0 | 0.000 | 0.051 | 0.000 | 0.000 |
| OTITIS MEDIA | 0 | 5 | 0 | 1 | 0.000 | 0.051 | 0.000 | 0.017 |
| VASCULITIS | 0 | 5 | 0 | 2 | 0.000 | 0.051 | 0.000 | 0.033 |
| LEUKEMIA | 0 | 4 | 0 | 2 | 0.000 | 0.041 | 0.000 | 0.033 |
| MULTIPLE MYELOMA | 0 | 4 | 1 | 2 | 0.000 | 0.041 | 0.017 | 0.033 |
| NEPHROSIS | 0 | 4 | 0 | 0 | 0.000 | 0.041 | 0.000 | 0.000 |
| OPTIC NEURITIS | 0 | 4 | 0 | 0 | 0.000 | 0.041 | 0.000 | 0.000 |
| RHINITIS | 0 | 4 | 0 | 0 | 0.000 | 0.041 | 0.000 | 0.000 |
| SEVERE COMBINED IMMUNODEFICIENCY | 0 | 4 | 0 | 0 | 0.000 | 0.041 | 0.000 | 0.000 |
| TRACHEITIS | 0 | 4 | 1 | 0 | 0.000 | 0.041 | 0.017 | 0.000 |
| AVIAN LEUKOSIS | 0 | 3 | 0 | 0 | 0.000 | 0.031 | 0.000 | 0.000 |
| CLASSICAL SWINE FEVER | 0 | 3 | 0 | 0 | 0.000 | 0.031 | 0.000 | 0.000 |
| COCCIDIOSIS | 0 | 3 | 0 | 0 | 0.000 | 0.031 | 0.000 | 0.000 |
| GLOMERULONEPHRITIS | 0 | 3 | 0 | 2 | 0.000 | 0.031 | 0.000 | 0.033 |
| HYDROCEPHALUS | 0 | 3 | 1 | 0 | 0.000 | 0.031 | 0.017 | 0.000 |
| LEPTOSPIROSIS | 0 | 3 | 0 | 0 | 0.000 | 0.031 | 0.000 | 0.000 |
| LIVER CIRRHOSIS | 0 | 3 | 0 | 1 | 0.000 | 0.031 | 0.000 | 0.017 |
| MALIGNANT GLIOMA | 0 | 3 | 0 | 0 | 0.000 | 0.031 | 0.000 | 0.000 |
| MENINGOENCEPHALITIS | 0 | 3 | 3 | 1 | 0.000 | 0.031 | 0.050 | 0.017 |
| NEURILEMMOMA | 0 | 3 | 0 | 0 | 0.000 | 0.031 | 0.000 | 0.000 |
| SIALADENITIS | 0 | 3 | 0 | 0 | 0.000 | 0.031 | 0.000 | 0.000 |
| STOMATITIS | 0 | 3 | 0 | 0 | 0.000 | 0.031 | 0.000 | 0.000 |
| ADENOCARCINOMA | 0 | 2 | 2 | 1 | 0.000 | 0.020 | 0.033 | 0.017 |
| ADENOMA | 0 | 2 | 0 | 2 | 0.000 | 0.020 | 0.000 | 0.033 |
| AMYLOIDOSIS | 0 | 2 | 0 | 0 | 0.000 | 0.020 | 0.000 | 0.000 |
| BALKAN NEPHROPATHY | 0 | 2 | 0 | 0 | 0.000 | 0.020 | 0.000 | 0.000 |
| BRAIN EDEMA | 0 | 2 | 0 | 2 | 0.000 | 0.020 | 0.000 | 0.033 |
| CONJUNCTIVITIS | 0 | 2 | 0 | 4 | 0.000 | 0.020 | 0.000 | 0.066 |
| CYSTIC FIBROSIS | 0 | 2 | 0 | 2 | 0.000 | 0.020 | 0.000 | 0.033 |
| FOWLPOX | 0 | 2 | 5 | 0 | 0.000 | 0.020 | 0.084 | 0.000 |
| GIARDIASIS | 0 | 2 | 0 | 0 | 0.000 | 0.020 | 0.000 | 0.000 |
| GINGIVITIS | 0 | 2 | 0 | 0 | 0.000 | 0.020 | 0.000 | 0.000 |
| HYPERGAMMAGLOBULINEMIA | 0 | 2 | 0 | 0 | 0.000 | 0.020 | 0.000 | 0.000 |
| LARYNGITIS | 0 | 2 | 0 | 0 | 0.000 | 0.020 | 0.000 | 0.000 |
| MUMPS | 0 | 2 | 2 | 2 | 0.000 | 0.020 | 0.033 | 0.033 |
| OTITIS MEDIA WITH EFFUSION | 0 | 2 | 0 | 0 | 0.000 | 0.020 | 0.000 | 0.000 |
| RETINITIS | 0 | 2 | 0 | 0 | 0.000 | 0.020 | 0.000 | 0.000 |
| SUBACUTE SCLEROSING PANENCEPHALITIS | 0 | 2 | 0 | 0 | 0.000 | 0.020 | 0.000 | 0.000 |
| TENOSYNOVITIS | 0 | 2 | 0 | 0 | 0.000 | 0.020 | 0.000 | 0.000 |
| UVEITIS | 0 | 2 | 0 | 0 | 0.000 | 0.020 | 0.000 | 0.000 |
| ACUTE CHEST SYNDROME | 0 | 1 | 0 | 0 | 0.000 | 0.010 | 0.000 | 0.000 |
| AORTIC ANEURYSM | 0 | 1 | 0 | 1 | 0.000 | 0.010 | 0.000 | 0.017 |
| ARTHRITIS | 0 | 1 | 0 | 0 | 0.000 | 0.010 | 0.000 | 0.000 |
| BRONCHIECTASIS | 0 | 1 | 0 | 0 | 0.000 | 0.010 | 0.000 | 0.000 |
| CHORIORETINITIS | 0 | 1 | 0 | 0 | 0.000 | 0.010 | 0.000 | 0.000 |
| CORONARY THROMBOSIS | 0 | 1 | 0 | 1 | 0.000 | 0.010 | 0.000 | 0.017 |
| CYSTICERCOSIS | 0 | 1 | 0 | 0 | 0.000 | 0.010 | 0.000 | 0.000 |
| DIABETIC KETOACIDOSIS | 0 | 1 | 0 | 4 | 0.000 | 0.010 | 0.000 | 0.066 |
| DIROFILARIASIS | 0 | 1 | 0 | 0 | 0.000 | 0.010 | 0.000 | 0.000 |
| DYSPEPSIA | 0 | 1 | 0 | 1 | 0.000 | 0.010 | 0.000 | 0.017 |
| ENTEROBIASIS | 0 | 1 | 0 | 0 | 0.000 | 0.010 | 0.000 | 0.000 |
| EOSINOPHILIA | 0 | 1 | 0 | 1 | 0.000 | 0.010 | 0.000 | 0.017 |
| FIBROSARCOMA | 0 | 1 | 0 | 0 | 0.000 | 0.010 | 0.000 | 0.000 |
| GAS GANGRENE | 0 | 1 | 0 | 0 | 0.000 | 0.010 | 0.000 | 0.000 |
| GLIOBLASTOMA MULTIFORME | 0 | 1 | 0 | 0 | 0.000 | 0.010 | 0.000 | 0.000 |
| GLUCOSEPHOSPHATE DEHYDROGENASE DEFICIENCY | 0 | 1 | 0 | 0 | 0.000 | 0.010 | 0.000 | 0.000 |
| HEPATIC ENCEPHALOPATHY | 0 | 1 | 0 | 0 | 0.000 | 0.010 | 0.000 | 0.000 |
| HEPATITIS E | 0 | 1 | 0 | 0 | 0.000 | 0.010 | 0.000 | 0.000 |
| HYPERCHOLESTEROLEMIA | 0 | 1 | 0 | 1 | 0.000 | 0.010 | 0.000 | 0.017 |
| HYPERINSULINISM | 0 | 1 | 0 | 0 | 0.000 | 0.010 | 0.000 | 0.000 |
| HYPOGLYCEMIA | 0 | 1 | 0 | 1 | 0.000 | 0.010 | 0.000 | 0.017 |
| INFECTIOUS BOVINE RHINOTRACHEITIS | 0 | 1 | 0 | 0 | 0.000 | 0.010 | 0.000 | 0.000 |
| INFECTIOUS MONONUCLEOSIS | 0 | 1 | 0 | 0 | 0.000 | 0.010 | 0.000 | 0.000 |
| LEPROSY | 0 | 1 | 0 | 0 | 0.000 | 0.010 | 0.000 | 0.000 |
| LYMPHADENITIS | 0 | 1 | 0 | 0 | 0.000 | 0.010 | 0.000 | 0.000 |
| MELANOMA | 0 | 1 | 0 | 2 | 0.000 | 0.010 | 0.000 | 0.033 |
| MENINGITIS | 0 | 1 | 0 | 1 | 0.000 | 0.010 | 0.000 | 0.017 |
| PANOPHTHALMITIS | 0 | 1 | 0 | 0 | 0.000 | 0.010 | 0.000 | 0.000 |
| PANUVEITIS | 0 | 1 | 0 | 0 | 0.000 | 0.010 | 0.000 | 0.000 |
| PARAPLEGIA | 0 | 1 | 0 | 0 | 0.000 | 0.010 | 0.000 | 0.000 |
| PARASITIC INFECTIOUS DISEASE | 0 | 1 | 0 | 0 | 0.000 | 0.010 | 0.000 | 0.000 |
| PERICARDITIS | 0 | 1 | 0 | 0 | 0.000 | 0.010 | 0.000 | 0.000 |
| PHLEBITIS | 0 | 1 | 0 | 0 | 0.000 | 0.010 | 0.000 | 0.000 |
| PLEURISY | 0 | 1 | 0 | 0 | 0.000 | 0.010 | 0.000 | 0.000 |
| PROTEIN DEFICIENCY | 0 | 1 | 0 | 0 | 0.000 | 0.010 | 0.000 | 0.000 |
| PROTEIN-ENERGY MALNUTRITION | 0 | 1 | 1 | 0 | 0.000 | 0.010 | 0.017 | 0.000 |
| RHABDOMYOSARCOMA | 0 | 1 | 0 | 0 | 0.000 | 0.010 | 0.000 | 0.000 |
| SCHISTOSOMIASIS | 0 | 1 | 0 | 0 | 0.000 | 0.010 | 0.000 | 0.000 |
| SINUSITIS | 0 | 1 | 0 | 0 | 0.000 | 0.010 | 0.000 | 0.000 |
| STATUS ASTHMATICUS | 0 | 1 | 0 | 0 | 0.000 | 0.010 | 0.000 | 0.000 |
| THEILERIASIS | 0 | 1 | 0 | 0 | 0.000 | 0.010 | 0.000 | 0.000 |
| THYMOMA | 0 | 1 | 0 | 0 | 0.000 | 0.010 | 0.000 | 0.000 |
| VACCINIA | 0 | 1 | 4 | 0 | 0.000 | 0.010 | 0.067 | 0.000 |
| VITAMIN A DEFICIENCY | 0 | 1 | 0 | 0 | 0.000 | 0.010 | 0.000 | 0.000 |
| WASTING SYNDROME | 0 | 1 | 0 | 0 | 0.000 | 0.010 | 0.000 | 0.000 |
| CANDIDIASIS | 0 | 0 | 1 | 0 | 0.000 | 0.000 | 0.017 | 0.000 |
| ENDOMETRITIS | 0 | 0 | 1 | 0 | 0.000 | 0.000 | 0.017 | 0.000 |
| EQUINE INFECTIOUS ANEMIA | 0 | 0 | 1 | 0 | 0.000 | 0.000 | 0.017 | 0.000 |
| GUILLAIN-BARRE SYNDROME | 0 | 0 | 1 | 12 | 0.000 | 0.000 | 0.017 | 0.199 |
| LEISHMANIASIS | 0 | 0 | 1 | 0 | 0.000 | 0.000 | 0.017 | 0.000 |
| REYE SYNDROME | 0 | 0 | 1 | 0 | 0.000 | 0.000 | 0.017 | 0.000 |
| BRAIN ISCHEMIA | 0 | 0 | 0 | 16 | 0.000 | 0.000 | 0.000 | 0.265 |
| VITAMIN D DEFICIENCY | 0 | 0 | 0 | 5 | 0.000 | 0.000 | 0.000 | 0.083 |
| APPENDICITIS | 0 | 0 | 0 | 4 | 0.000 | 0.000 | 0.000 | 0.066 |
| DEMENTIA | 0 | 0 | 0 | 4 | 0.000 | 0.000 | 0.000 | 0.066 |
| ANTIPHOSPHOLIPID SYNDROME | 0 | 0 | 0 | 3 | 0.000 | 0.000 | 0.000 | 0.050 |
| INFERTILITY | 0 | 0 | 0 | 3 | 0.000 | 0.000 | 0.000 | 0.050 |
| ISCHEMIA | 0 | 0 | 0 | 3 | 0.000 | 0.000 | 0.000 | 0.050 |
| SCHIZOPHRENIA | 0 | 0 | 0 | 3 | 0.000 | 0.000 | 0.000 | 0.050 |
| ALOPECIA | 0 | 0 | 0 | 2 | 0.000 | 0.000 | 0.000 | 0.033 |
| AORTIC VALVE STENOSIS | 0 | 0 | 0 | 2 | 0.000 | 0.000 | 0.000 | 0.033 |
| FETAL DISTRESS | 0 | 0 | 0 | 2 | 0.000 | 0.000 | 0.000 | 0.033 |
| HEAD AND NECK SQUAMOUS CELL CARCINOMA | 0 | 0 | 0 | 2 | 0.000 | 0.000 | 0.000 | 0.033 |
| HYPERTHYROIDISM | 0 | 0 | 0 | 2 | 0.000 | 0.000 | 0.000 | 0.033 |
| HYPOCHONDRIASIS | 0 | 0 | 0 | 2 | 0 | 0 | 0 | 0 |
| MYASTHENIA GRAVIS | 0 | 0 | 0 | 2 | 0 | 0 | 0 | 0 |
| PNEUMATOSIS CYSTOIDES INTESTINALIS | 0 | 0 | 0 | 2 | 0 | 0 | 0 | 0 |
| PROTEINURIA | 0 | 0 | 0 | 2 | 0 | 0 | 0 | 0 |
| STRABISMUS | 0 | 0 | 0 | 2 | 0 | 0 | 0 | 0 |
| THALASSEMIA | 0 | 0 | 0 | 2 | 0 | 0 | 0 | 0 |
| ANGIOEDEMA | 0 | 0 | 0 | 1 | 0 | 0 | 0 | 0 |
| AORTIC VALVE INSUFFICIENCY | 0 | 0 | 0 | 1 | 0 | 0 | 0 | 0 |
| ARTERIOSCLEROSIS OBLITERANS | 0 | 0 | 0 | 1 | 0 | 0 | 0 | 0 |
| ATHEROSCLEROSIS | 0 | 0 | 0 | 1 | 0 | 0 | 0 | 0 |
| AUTISTIC DISORDER | 0 | 0 | 0 | 1 | 0 | 0 | 0 | 0 |
| AZOOSPERMIA | 0 | 0 | 0 | 1 | 0 | 0 | 0 | 0 |
| BENIGN MESOTHELIOMA | 0 | 0 | 0 | 1 | 0 | 0 | 0 | 0 |
| CALCINOSIS | 0 | 0 | 0 | 1 | 0 | 0 | 0 | 0 |
| CARDIAC TAMPONADE | 0 | 0 | 0 | 1 | 0 | 0 | 0 | 0 |
| CELLULITIS | 0 | 0 | 0 | 1 | 0 | 0 | 0 | 0 |
| COCCIDIOIDOMYCOSIS | 0 | 0 | 0 | 1 | 0 | 0 | 0 | 0 |
| COMMON VARIABLE IMMUNODEFICIENCY | 0 | 0 | 0 | 1 | 0 | 0 | 0 | 0 |
| CONJUNCTIVAL PTERYGIUM | 0 | 0 | 0 | 1 | 0 | 0 | 0 | 0 |
| CYCLOTHYMIC DISORDER | 0 | 0 | 0 | 1 | 0 | 0 | 0 | 0 |
| URTICARIA | 0 | 0 | 0 | 1 | 0 | 0 | 0 | 0 |
| DENTAL CARIES | 0 | 0 | 0 | 1 | 0 | 0 | 0 | 0 |
| DIABETES INSIPIDUS | 0 | 0 | 0 | 1 | 0 | 0 | 0 | 0 |
| ENDOCARDITIS | 0 | 0 | 0 | 1 | 0 | 0 | 0 | 0 |
| ENDOPHTHALMITIS | 0 | 0 | 0 | 1 | 0 | 0 | 0 | 0 |
| FACIAL PARALYSIS | 0 | 0 | 0 | 1 | 0 | 0 | 0 | 0 |
| FRONTAL SINUSITIS | 0 | 0 | 0 | 1 | 0 | 0 | 0 | 0 |
| GLYCOGEN STORAGE DISEASE II | 0 | 0 | 0 | 1 | 0 | 0 | 0 | 0 |
| HEPATIC VEIN THROMBOSIS | 0 | 0 | 0 | 1 | 0 | 0 | 0 | 0 |
| HIDRADENITIS SUPPURATIVA | 0 | 0 | 0 | 1 | 0 | 0 | 0 | 0 |
| HYPERPARATHYROIDISM | 0 | 0 | 0 | 1 | 0 | 0 | 0 | 0 |
| HYPERURICEMIA | 0 | 0 | 0 | 1 | 0 | 0 | 0 | 0 |
| HYPOTHYROIDISM | 0 | 0 | 0 | 1 | 0 | 0 | 0 | 0 |
| HYPOTRICHOSIS | 0 | 0 | 0 | 1 | 0 | 0 | 0 | 0 |
| INAPPROPRIATE ADH SYNDROME | 0 | 0 | 0 | 1 | 0 | 0 | 0 | 0 |
| INTESTINAL OBSTRUCTION | 0 | 0 | 0 | 1 | 0 | 0 | 0 | 0 |
| INTRACRANIAL ANEURYSM | 0 | 0 | 0 | 1 | 0 | 0 | 0 | 0 |
| INTRACRANIAL HYPERTENSION | 0 | 0 | 0 | 1 | 0 | 0 | 0 | 0 |
| IRRITABLE BOWEL SYNDROME | 0 | 0 | 0 | 1 | 0 | 0 | 0 | 0 |
| KERATITIS | 0 | 0 | 0 | 1 | 0 | 0 | 0 | 0 |
| KERATOCONJUNCTIVITIS | 0 | 0 | 0 | 1 | 0 | 0 | 0 | 0 |
| KYPHOSIS | 0 | 0 | 0 | 1 | 0 | 0 | 0 | 0 |
| LONG QT SYNDROME | 0 | 0 | 0 | 1 | 0 | 0 | 0 | 0 |
| LUNG ADENOCARCINOMA | 0 | 0 | 0 | 1 | 0 | 0 | 0 | 0 |
| LYMPHEDEMA | 0 | 0 | 0 | 1 | 0 | 0 | 0 | 0 |
| MILLER FISHER SYNDROME | 0 | 0 | 0 | 1 | 0 | 0 | 0 | 0 |
| MYOTONIC DYSTROPHY | 0 | 0 | 0 | 1 | 0 | 0 | 0 | 0 |
| NEURITIS | 0 | 0 | 0 | 1 | 0 | 0 | 0 | 0 |
| NEUROMYELITIS OPTICA | 0 | 0 | 0 | 1 | 0 | 0 | 0 | 0 |
| ORBITAL CELLULITIS | 0 | 0 | 0 | 1 | 0 | 0 | 0 | 0 |
| PANIC DISORDER | 0 | 0 | 0 | 1 | 0 | 0 | 0 | 0 |
| PERICARDIAL EFFUSION | 0 | 0 | 0 | 1 | 0 | 0 | 0 | 0 |
| PITUITARY APOPLEXY | 0 | 0 | 0 | 1 | 0 | 0 | 0 | 0 |
| PRE-ECLAMPSIA | 0 | 0 | 0 | 1 | 0 | 0 | 0 | 0 |
| PREDIABETES SYNDROME | 0 | 0 | 0 | 1 | 0 | 0 | 0 | 0 |
| PREGNANCY IN DIABETICS | 0 | 0 | 0 | 1 | 0 | 0 | 0 | 0 |
| PULPITIS | 0 | 0 | 0 | 1 | 0 | 0 | 0 | 0 |
| PURPURA | 0 | 0 | 0 | 1 | 0 | 0 | 0 | 0 |
| QUADRIPLEGIA | 0 | 0 | 0 | 1 | 0 | 0 | 0 | 0 |
| SCARLET FEVER | 0 | 0 | 0 | 1 | 0 | 0 | 0 | 0 |
| SIMIAN ACQUIRED IMMUNODEFICIENCY SYNDROME | 0 | 0 | 0 | 1 | 0 | 0 | 0 | 0 |
| SJOGRENS SYNDROME | 0 | 0 | 0 | 1 | 0 | 0 | 0 | 0 |
| THROMBOCYTOSIS | 0 | 0 | 0 | 1 | 0 | 0 | 0 | 0 |
| THYROID NODULE | 0 | 0 | 0 | 1 | 0 | 0 | 0 | 0 |

*only diseases with >1 occurrences are shown
